# Migration of an Inferior Vena Cava (IVC) Filter Into the Intrahepatic IVC: A Case Report

**DOI:** 10.7759/cureus.26308

**Published:** 2022-06-24

**Authors:** Alexis Gazda, Marc Squillante

**Affiliations:** 1 Department of Emergency Medicine, University of Illinois College of Medicine - Peoria, Peoria, USA

**Keywords:** ivc filter retrieval, ivc filter complication, interventional radiology, intrahepatic ivc, ivc filter migration

## Abstract

The use of Inferior Vena Cava (IVC) filters is increasing for patients who cannot otherwise receive anticoagulation for a deep vein thrombosis (DVT) or pulmonary embolism (PE). In this case, a 60-year-old man presenting with abdominal pain was found to have migration of his IVC filter into his intrahepatic IVC. Interventional radiology (IR) and vascular surgery were consulted as the migrated IVC filter was felt to be the likely cause of the patient’s pain. Consideration was given to transferring the patient to a liver transplant center to retrieve the migrated filter. When patients with a history of IVC filter placement present to the emergency department, a high index of suspicion for IVC filter complication should be maintained and there should be a low threshold for ordering CT imaging. If a severe IVC filter complication is discovered, patients may ultimately require transfer to a center with hepatic surgery capability for definitive treatment given the complex vasculature involved.

## Introduction

Inferior vena cava (IVC) filters exist for the treatment of venous thromboembolism for those who otherwise cannot receive anticoagulation. With the increase in the diagnosis of deep vein thrombosis (DVT) and pulmonary embolism (PE) over the past several decades, there has also been an increase in the number of IVC filters placed [1]. However, these filters have complications of their own including complications of insertion, caval wall penetration, obstruction of the IVC, and filter migration [1,2].

X-rays and CT scans obtained in the emergency department (ED) can illustrate IVC filter-related problems. Subsequently, if an IVC filter-related problem is identified, that imaging can help guide management including conservative, interventional, or surgical [1]. Although an IVC filter-related problem may not be the primary purpose for ordering a CT scan in the ED, it is important for the emergency physician to consider an IVC filter-related problem in the differential diagnosis. The emergency physician should be aware of possible complications related to the IVC filter and anticipate management of these in order to provide proper patient care in the ED, facilitate timely patient dispositions, and obtain appropriate consultations.

## Case presentation

Our patient was a 60-year-old male who had a glioblastoma diagnosed in 2019 necessitating craniotomies in April 2019 and August 2020. He was admitted in September 2020 (two months prior to the emergency department visit) for headaches and craniotomy incisional leakage. He had an external ventricular drain placed during that admission that was complicated by intraventricular hemorrhage. During that admission, he was also noted to have bilateral DVTs. However, because he had recently undergone cranial surgery complicated by intraventricular hemorrhage, an IVC filter was placed rather than placing the patient on anticoagulation. Interventional Radiology (IR) placed a VenaTech (B. Braun Interventional Systems, Inc, Bethlehem, PA, USA) permanent IVC filter on October 20, 2020. He was clinically improving afterwards and was discharged a few days later. The patient was admitted to an acute rehab facility in November 2020 as he had lost some independence and mobility after his extensive neurosurgical course and multiple admissions.

The patient presented to the emergency department on the day after discharge from his November 2020 admission with a chief complaint of abdominal pain. He developed right lower quadrant abdominal pain on the day of discharge from that admission. He described it as a twisting pain that worsened with movement and radiated to his right flank. His pain had intensified since his discharge, prompting him to come to the ED for further evaluation. He had no associated fevers, vomiting, or diarrhea. The patient had no previous history of any abdominal surgeries, history of kidney stones, or recurrent urinary tract infections. When the patient presented to the ED he was tachycardic and afebrile with otherwise normal vital signs. On exam, he had tenderness to palpation of the right upper and lower quadrants of his abdomen with voluntary guarding. His abdomen was soft and he had no rebound tenderness. His physical exam was otherwise normal.

His lab work, including a complete blood count (CBC), comprehensive metabolic panel (CMP), lipase, lactate, and urinalysis was unremarkable. A COVID test was negative.

**Figure 1:**
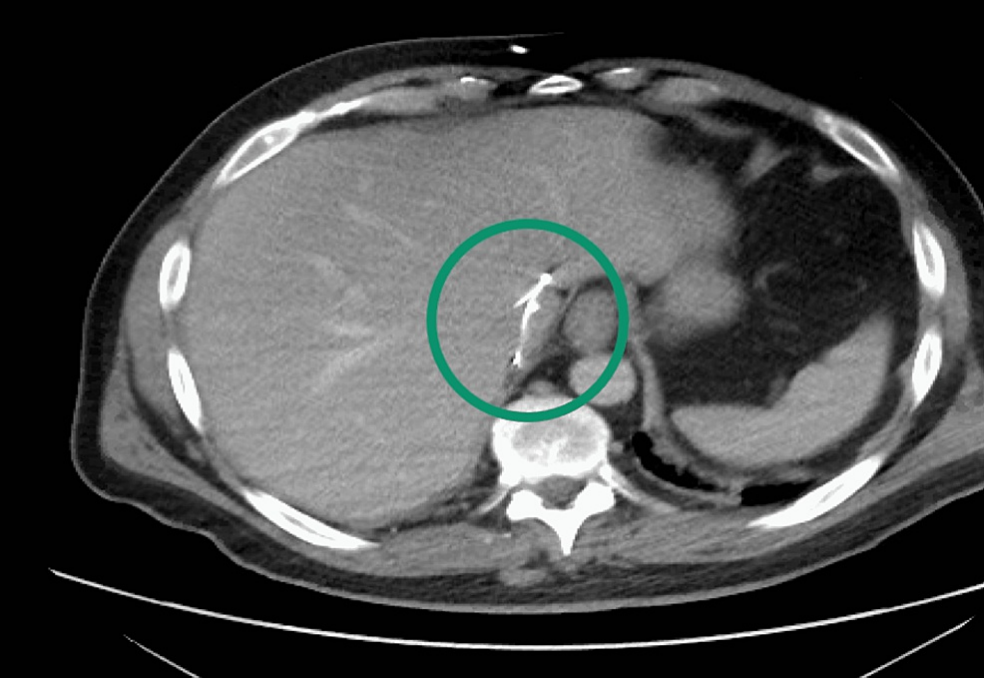
Cross-sectional view of IVC filter in intrahepatic IVC IVC: Inferior Vena Cava

**Figure 2:**
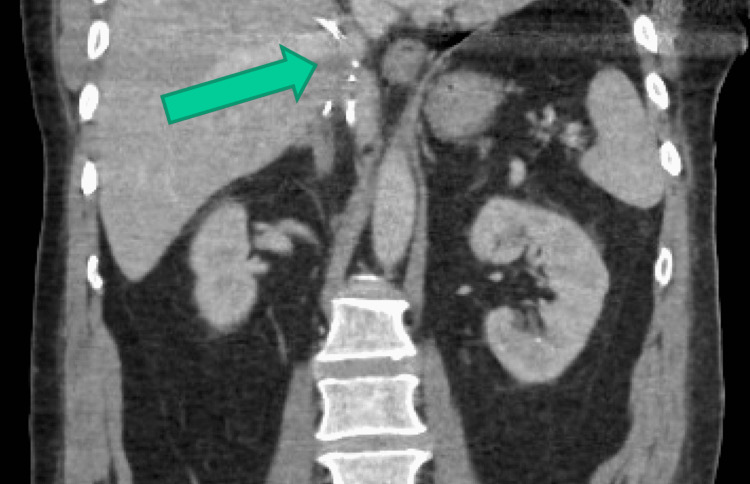
Coronal view of IVC filter in intrahepatic IVC IVC: Inferior Vena Cava

Throughout his workup in the ED his pain was so severe that he required multiple doses of IV narcotics for pain control. Given the history of his recent inferior vena cava filter placement, severity and acuity of his pain, and unrevealing labs, a contrasted CT of the patient’s abdomen and pelvis was ordered for further evaluation. The CT showed that the patient’s IVC filter had migrated from its original location in the infrarenal IVC to the intrahepatic IVC (Figures 1-2). It was also malaligned and rotated. There were also extensive, nonoccluding DVTs extending from the IVC filter through the common femoral veins. Additionally, there was a nonspecific ground-glass opacity in the patient’s right lower lung. No other acute abnormalities were seen on the CT scan.

Radiology contacted us shortly after the CT scan was completed and expressed concern for a PE given the extensive clot burden behind the filter, the patient’s pain, and the right lower lobe ground-glass opacity. Radiology recommended a CT angiogram of the patient’s chest and IR consultation for the migrated IVC filter, as it was felt to be the likely source of the patient’s abdominal pain. IR stated that placement and retrieval of IVC filters is done exclusively by them with few exceptions. However, when severe complications arise and the IR team is not able to retrieve them safely, then surgical intervention should be considered. IR further explained that because migration to the intrahepatic IVC is rare, vascular surgery should be consulted for possible filter removal. With the location of the patient’s IVC filter and the complex vasculature involved, vascular surgery recommended potentially transferring the patient to a liver transplant center if surgery is required to remove the IVC filter.

**Figure 3:**
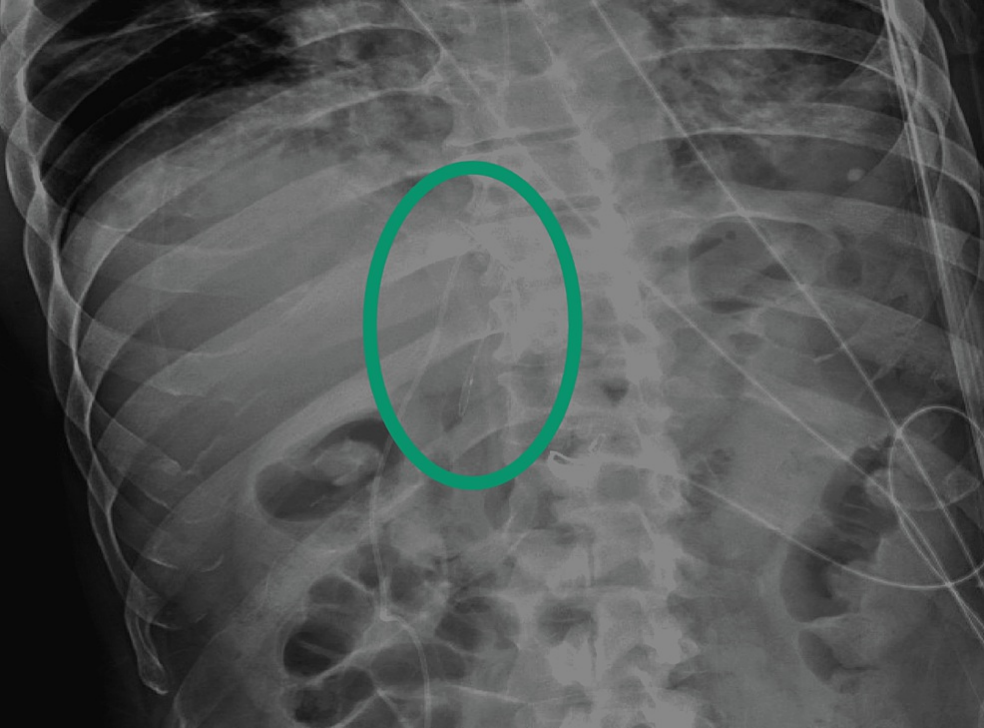
Abdominal X-ray showing the IVC filter IVC: Inferior Vena Cava

The patient was ultimately admitted to the hospital for pain control with IR consultation. He was started on intravenous heparin for the possible PE after consultation with neurosurgery. A plain abdominal film was performed the next morning which showed that the filter had not migrated from the day of admission (Figure 3). There were discussions with several IR attendings about removing the filter. However, given the patient’s extensive clot burden, IR recommended continuing anticoagulation and removing the filter at a later date if the patient’s pain persisted. During the admission, the patient had a CT angiogram of his chest, which showed bilateral pulmonary emboli that had likely gotten past the IVC filter. The patient was continued on heparin throughout the admission and was discharged on rivaroxaban.

A month after discharge, a repeat abdominal CT showed the IVC filter still in the intrahepatic IVC. The clot burden distal to the filter was improved. The patient followed up with IR as an outpatient as his pain persisted after discharge. IR ultimately was able to retrieve the IVC filter three months after the migration was first discovered. The patient’s pain resolved after the IVC filter was removed and he was continued on oral anticoagulation.

## Discussion

With the increased use of IVC filters, there is also an increase in the number of patients presenting with IVC filter complications. IVC filter complications include difficulties with insertion, filter migration, IVC occlusion, vessel perforation, and increased risk of DVT [1-3]. Furthermore, these complications are associated with the amount of time they are in place [3]. In 2010 the FDA issued a communication recommending filter removal as soon as possible once its use is no longer indicated, as there is increased morbidity and mortality with retrieval. Daye and Walker recommend that the IVC filter be removed within two months of filter placement or as soon as venous thromboembolism is negligible [4]. Forty to sixty percent of removals can be technically challenging. If the filter is embedded or tilted, then advanced techniques are subsequently needed for removal [4]. Retrieval rate for permanent filters is low, ranging from 6-24% [5].

IVC filter migration is a particularly worrisome complication. A comprehensive review of IVC filters was done by Li et al. in 2020 [5]. Filter migration is defined as greater than 2 cm displacement of the IVC filter from its original position. Rates of migration vary anywhere from 0-11.8%. When patients become symptomatic from filter migration definitive management is needed, often including filter removal. IVC filters are normally placed in the infrarenal IVC. Reports have been made of IVC filter migration to the heart, pulmonary arteries, intrahepatic IVC, hepatic veins, renal veins, iliac veins, and superior vena cava (SVC) [1, 6-7].

A review article by Wu et al. in 2014 [7] also demonstrated the severity of IVC filter migration. Retrieval of an IVC filter can be extremely technically difficult and requires thoughtful planning; despite careful planning, removal of the migrated filter can be very complicated and often unsuccessful. Removal is also associated with fragmentation, embolization, and increase in patient morbidity and mortality. Thirty-eight cases discussed for this review showed filter migration to the SVC, brachiocephalic venous branches, right atrium, right jugular vein, and iliac venous confluence. Twenty-three of the 38 cases required surgical consultation. Eighteen of these 38 cases showed migration of the filter. Seven of the 18 migrated filters had retrieval attempted and only four out of the 18 cases had a successful retrieval. All four cases were complicated by fracture of the filter.

Unfortunately, our patient’s presenting symptom of pain ultimately revealing an IVC filter complication is not uncommon. Georgiou et al. [1] reviewed multiple cases of IVC abnormalities identified on CT scans and discussed an IVC filter placed in a pregnant woman that ultimately required removal after it caused her to have persistent pain in her right upper quadrant. Another patient in Georgiou’s review showed an incidental finding of an IVC filter in the patient’s intrahepatic IVC, although that patient did not complain of pain. A case report in 2015 discussed a patient with years of abdominal pain who was found to have their IVC filter perforated through the duodenum [8]. A case report in 2021 discussed a patient presenting with chest pain who had an IVC filter piece fractured and migrated into her lung [9]. Friedell et al. reported on a patient with an IVC filter presenting with chest pain; that patient’s IVC filter had migrated to the pulmonary artery [6].

While there have been documented cases of successful removal of IVC filter fragments from multiple sites including the IVC, aorta, pulmonary artery and right ventricle, the success rate for these removals was highly variable, with rates ranging from 50-100% [5]. These removals were also associated with complications including further embolization of the fragment during attempted retrieval, bacteremia, pulmonary infarction, perioperative stroke, and one case of cardiac tamponade [10]. Limited literature is available on the removal of the entire filter that has migrated into the intrahepatic IVC. A study by Neuerburg et al. [11] performed retrieval of IVC filter fragments into the lumbar and intrahepatic segments of the IVC in dogs in 2001. They were able to remove 20 of the 21 IVC filters placed into these segments of the IVC two weeks after being placed. They could not remove the last filter as it was tilted in the intrahepatic IVC and it was deemed too technically difficult and dangerous to remove. With these studies in mind, the recommendation from the vascular surgeon to potentially transfer our patient to a facility capable of performing hepatic surgery (if percutaneous techniques were not successful given the technical difficulty in removing our patient’s malaligned IVC filter in his intrahepatic IVC), was reasonable.

History is important for the emergency physician in evaluating these patients. Any patient with a history of a DVT or PE should be asked whether they have an IVC filter in place. If so, it is important for the ED physician to consider this when determining patient differentials and workup. In patients with abdominal or chest pain with a known IVC filter in place, the emergency physician should maintain a high index of suspicion for an IVC filter-related problem, particularly if that IVC filter has been in place for several months or years. Emergency physicians should have a low threshold for ordering a CT scan with contrast to further evaluate a patient with an IVC filter in place that presents with pain. CT scan may help determine the cause of the patient’s pain and may help with any preprocedural planning if the IVC filter is the culprit.

## Conclusions

For any patient with a history of a DVT or PE, the emergency physician should inquire as to whether the patient has a current IVC filter, regardless of the patient’s chief complaint for that ED visit. Emergency physicians should also have a low threshold for ordering a CT scan for any patient with an IVC filter in place as there is a possibility that the IVC filter may be related to the patient’s complaint. In the event that the patient does have a complication involving the IVC filter, IR and vascular surgery should be consulted, and the patient may require transfer to a facility that is capable of performing the complex process of filter removal.
